# Association between residential proximity to major roadways and chronic multimorbidity among Chinese older adults: a nationwide cross-sectional study

**DOI:** 10.1186/s12877-024-04712-z

**Published:** 2024-01-29

**Authors:** Xuange Sun, Xu Liu, Xue Wang, Chang Pang, Zhihua Yin, Shuang Zang

**Affiliations:** 1 Department of Community Nursing, School of Nursing, China Medical University, No.77 Puhe Road, Shenyang North New Area, 110122 Shenyang, Liaoning Province China; 2 Department of General Practice, The Second Affiliated Hospital of Shenyang Medical College, No.20 Bei Jiu Road, Heping District, 110002 Shenyang, Liaoning Province China; 3 Department of epidemiology, School of Public Health, China Medical University, No.77 Puhe Road, Shenyang North New Area, 110122 Shenyang, Liaoning Province China

**Keywords:** Major roadway, Traffic exposure, Older adults, CLHLS, Multimorbidity, Chronic disease

## Abstract

**Background:**

Multiple negative health outcomes were linked to residential proximity to major roadways. Nevertheless, there is limited knowledge regarding the association between residential proximity to major roadways and chronic multimorbidity.

**Methods:**

We used data from the 2018 wave of the Chinese Longitudinal Healthy Longevity Survey, which included 12,214 individuals aged ≥ 60. We derived the residential proximity to major roadways from self-reported data, defining chronic multimorbidity as the presence of two or more concurrent chronic diseases. A binary logistic regression model was utilized to investigate the association between residential proximity to major roadways and chronic multimorbidity. The model accounted for some demographic features, socioeconomic conditions, social participation, and health conditions. Subsequently, we conducted subgroup analyses to examine potential interaction effects.

**Results:**

Residential proximity to major roadways was associated with chronic multimorbidity, even after adjusting for confounding factors. Compared with those living > 300 m from major roadways, the OR for those living 201-300 m, 101-200 m, 50-100 m, and < 50 m were increased. When subgroup analyses were conducted using a cutoff point of 200 m, the risk of chronic multimorbidity associated with residential proximity to major roadways was stronger in participants with education levels > 6 years (*P* = 0.017).

**Conclusion:**

Our findings provide important implications for improving residential area siting, transportation policies, and environmental regulations to reduce the risk of chronic multimorbidity caused by traffic-related exposure.

**Supplementary Information:**

The online version contains supplementary material available at 10.1186/s12877-024-04712-z.

## Introduction

Healthy aging is a crucial issue due to the demographic challenges posed by population aging, which has attracted significant attention globally [1]. Simultaneously, China has emerged as one of the countries undergoing the most rapid increase in the aging population around the world. As reported in 2022, the group of Chinese individuals aged 60 and over has reached 280 million, making up 19.8% of the overall population [2]. The characteristic of aging is marked by the simultaneous presence of numerous chronic conditions, which might make up multimorbidity.

In a nationwide study conducted in China, a prevalence rate of 49.64% was observed for multimorbidity among individuals aged 60 and over [3]. As a result, the presence of multiple chronic conditions has emerged as a significant concern for public health in China [4]. There exists substantial evidence indicating that chronic multimorbidity has a considerable impact on various aspects such as functional status or health-related quality of life [5, 6], health resource utilization [7], activities of daily living instrumental [8], even the disability [9], and mortality [10]. Given that the simultaneous occurrence of multiple chronic diseases is not accidental [11], identifying shared factors associated with multiple chronic diseases could optimize disease prevention strategies and conventional clinical intervention approaches.

Among the various factors associated with health outcomes in aging populations, the individuals’ living environment has emerged as a critical determinant, one of which is the residential proximity to major roadways [12]. An ever-growing body of evidence points to a notable link between residential proximity to major roadways and some chronic diseases, such as hypertension [13], stroke [14], and dementia [15]. However, current research solely concentrates on the association between residential proximity to major roadways and solitary chronic disease. Since chronic multimorbidity is more common in older adults, numerous causative factors from a single source can lead to multiple outcomes, and different diseases can impact or trigger one another. Therefore, it is necessary to clarify the association between residential proximity to major roadways and multimorbidity among older adults.

In addition, residential proximity to major roadways is a known source of several environmental exposures [16, 17], including traffic-related air pollution and noise. According to a UK study, air pollution could have a role in nearly every transition stage of the development of cardiometabolic multimorbidity [18]. Meanwhile, another study found that senior Mexican Americans who experience traffic noise have a higher chance of developing dementia in their later years and cognitive decline [19]. Nevertheless, these studies only reveal the association between a single type of traffic-related exposure factor and a certain kind of disease. On the other hand, these studies mentioned above were conducted in Western countries, given the variations in residential preferences and living environments, which may not be applicable to China [20]. Since in wealthier Western nations, more rigorous controls on motor vehicle emissions are implemented, possible variations in traffic-related hazard [21]. Consequently, this study utilizes residential proximity to major roadways as a comprehensive surrogate measure for traffic-related exposures mentioned above, aiming to investigate the association between traffic-related exposures and chronic multimorbidity in the specific context of China, which is one of the most populous countries and the second largest economy in the world.

## Methods

### Study population

The data of this study was derived from the 2018 wave of the Chinese Longitudinal Healthy Longevity Survey (CLHLS), which consists of a sequence of surveys carried out every 2 to 3 years since 1998. The survey employed a multi-stage disproportionate and targeted random sampling approach to guarantee the collection of representative and dependable data [22]. A random selection was used to choose half of the total cities (counties) from 22 out of the 31 provinces/autonomous regions/municipalities in mainland China. In addition, CLHLS oversamples the oldest individuals aged ≥ 80 years. Till 2018, 67.4% of participants in CLHLS were people aged ≥ 80 years [23]. Details of this database have been published elsewhere [24].

**Fig. 1:**
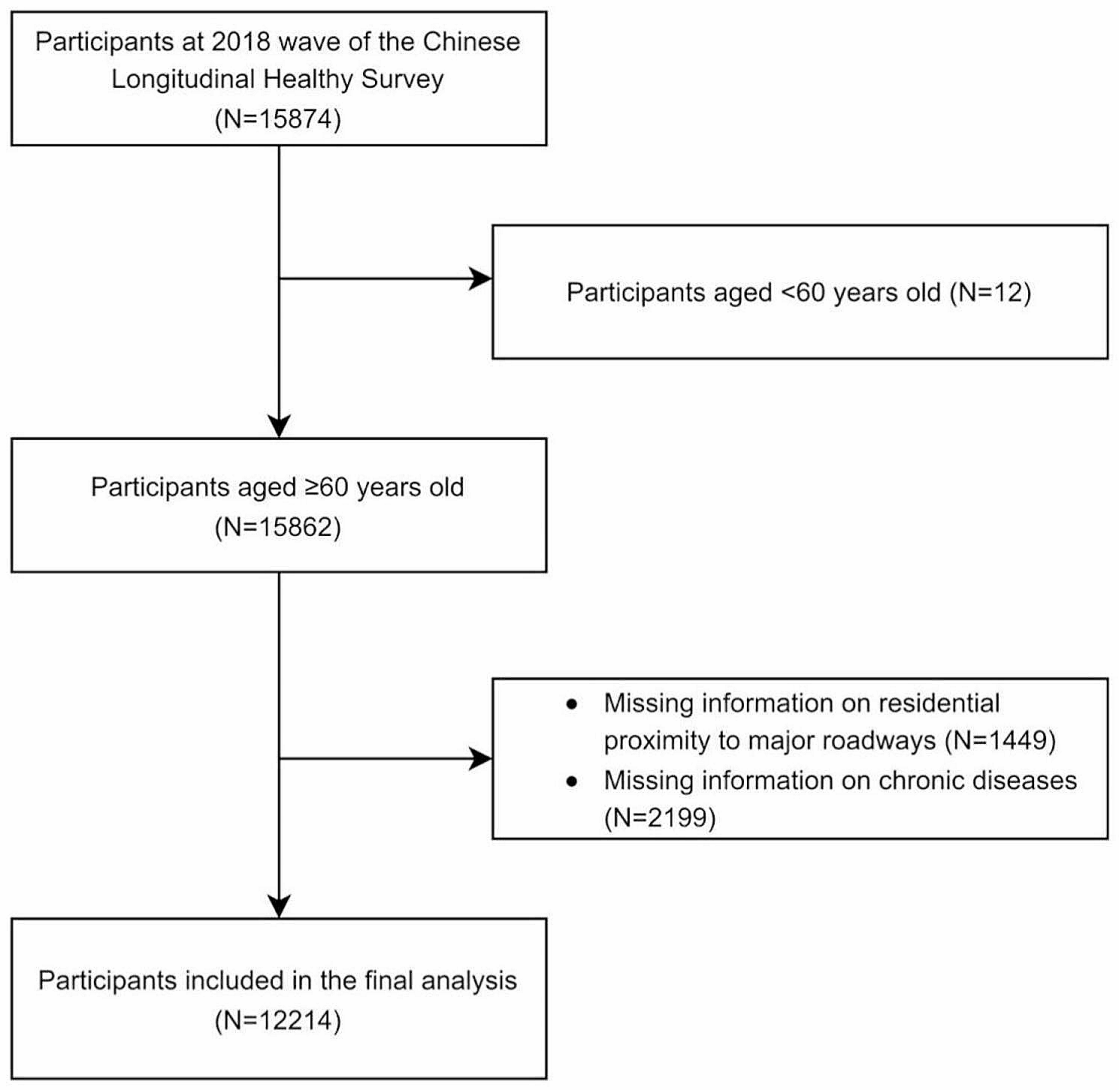
Flow chart inclusion of participants from the Chinese longitudinal healthy longevity survey

CLHLS collected data from older adults through in-person interviews with an internationally standardized questionnaire appropriate for Chinese cultural and social environment. The questionnaire covered demographic features, socioeconomic conditions, health conditions, psychological traits, and cognitive function [25]. In the 2018 wave of CLHLS, a total of 15,874 participants were assessed. According to the criteria of the World Health Organization, older adults are considered to be 60 years of age and over in developing countries and 65 years of age and over in developed countries [26]. As China is a developing country, participants younger than 60 years old were excluded from this study (*N* = 12). Additionally, participants with missing data on self-reported residential proximity to major roadways (*N* = 1449) and with any missing data on 17 chronic diseases (*N* = 2199) were excluded. The remaining analytical sample comprised 12,214 older adults (Fig. 1).

The CLHLS study (IRB00001052–13,074) conducted by the Ethics Committee of Peking University has been granted approval. Written consent forms were signed by all the participants.

### Residential proximity to major roadways

The major roadways are defined as “dual carriageways with at least four lanes.” To classify their present residential proximity to the closest major roadways, participants were asked to answer a question with five options: < 50 m, 50–100 m, 101–200 m, 201–300 m, and > 300 m.

### Definitions of chronic multimorbidity

Participants were asked, “Have you been diagnosed with the following diseases by a doctor?” Considering the high prevalence and high co-morbidity rate of diseases among older adults [27, 28], this study included 17 chronic diseases as the alternative answers, including heart disease, stroke, hypertension, diabetes, chronic lung disease (bronchiolitis, emphysema, asthma, pneumonia), tuberculosis, cancer, dyslipidemia, arthritis, peptic ulcer, cholecystitis and cholelithiasis, Parkinson’s disease, rheumatologic disease, chronic nephritis, hepatitis, dementia, epilepsy. Respondents answered “1 = yes” or “0 = no” for each item. When two or more of these 17 chronic diseases coexist in the same individual, it is called multimorbidity [29, 30].

### Covariates

We considered the following potential confounding variables in this study based on the previous studies [14, 31, 32].

#### Routine covariates

The basic demographic features included age, gender (male vs. female), area of residence (urban vs. rural), ethnicity (Han vs. other), and marital status (have a spouse vs. have no spouse). The socioeconomic conditions were education level (0 years, 1–6 years, and > 6 years) and annual household income (< 30,000 yuan, 30,000–50,000 yuan, and > 50,000 yuan). Health conditions included smoking status (yes vs. no), alcohol consumption (yes vs. no), physical activity (refers to purposeful fitness activities such as strolling, playing ball, running, and qigong) (yes vs. no), and self-rated health status (1 = very bad to 5 = very good).

#### Measurements

##### Depression symptoms

The Center for Epidemiologic Studies Depression Scale-10 (CES-D-10) is used to evaluate participants’ depression symptoms [33]. Each item is scored on a 4-point scale of 0–3, 0 = less than once during the past week, 1 = one to two days, 2 = three to four days, and 3 = five days and more. Summed scores of the CES-D-10 range from 0 to 30 points, with higher scores representing severer depression symptoms. Following prior studies [34, 35], a person who scores more than or equal to 10 is regarded as having existing depression symptoms. The Cronbach’s α for the CES-D-10 of this study was 0.732.

##### Anxiety symptoms

The Generalized Anxiety Disorder Scale-7 (GAD-7) is used to screen and assess the severity of anxiety symptoms [36]. Each item is scored on a 4-point scale of 0–3, 0 = not at all; 1 = days away; 2 = more than one week; 3 = almost daily. Summed scores of the GAD-7 range from 0 to 21 points, with higher scores representing severer anxiety symptoms. Following prior studies [37, 38], a person who scores more than or equal to 5 is regarded as having existing anxiety symptoms. The Cronbach’s α for the GAD-7 of this study was 0.919.

##### Cognitive impairment

The Chinese Mini-Mental State Examination (CMMSE) is used to assess participants’ cognitive function [39]. The CMMSE encompasses 24 items distributed across seven cognitive domains. These include orientation (allocated four points for temporal awareness, and one for spatial awareness); rapid food naming (enumerating as many food types within a minute, seven points); word repetition (three points); attention and mathematical processing (sequential subtraction of three from twenty, five points); figure duplication (one point); memory recall (remembering the previously mentioned three words, three points); and linguistic abilities (two points for object identification, one for sentence repetition, and three for auditory comprehension and implementation). Summed scores of the CMMSE range from 0 to 30, with higher scores indicating better cognitive function. Following prior studies [40, 41], a person who scores less than 18 is regarded as having cognitive impairment. The Cronbach’s α for the CMMSE of this study was 0.906.

### Statistical analyses

Firstly, the Kolmogorov-Smirnov test was used to assess the normality of continuous variables. The Q-Q plots suggested that the continuous variables did not follow a normal distribution. Secondly, descriptive analyses of the study participants were reported using the median (IQR) for continuous variables and frequency (%) distribution for categorical variables. To assess the differences among the five proximity categories (< 50 m, 50-100 m, 101-200 m, 201-300 m, and > 300 m), the Kruskal-Wallis tests were used for continuous variables, and chi-square tests were used for categorical variables. Finally, binary logistic regression models were performed to explore the association between residential proximity to major roadways with chronic multimorbidity. In the analysis, three models were employed: Model 1 was unadjusted; Model 2 was adjusted for age, gender, area of residence, ethnicity, and marital status; Model 3 was adjusted for age, gender, area of residence, ethnicity, marital status, education level, annual household income, smoking status, alcohol consumption, physical activity, depression symptoms, anxiety symptoms, cognitive impairment, and self-rated health status.

A study conducted in recent years pointed out that 200 m might be an ideal divide standard of residential exposure severity [42]. Therefore, we dichotomized residential proximity to major roadways (≤ 200 m, > 200 m) in subgroup analyses and the test for interaction. To identify potential modifying variables, stratified analyses were adopted for gender, area of residence, ethnicity, marital status, education level, annual household income, smoking status, alcohol consumption, physical activity, depression symptoms, anxiety symptoms, and cognitive impairment. All other covariables were adjusted except the stratified variable.

Three sensitivity analyses were also conducted. In the first sensitivity analysis, multiple imputation by chained equations, an iterative imputation procedure, was used to impute the missing values, and the imputed data were then used for analysis. In the second sensitivity analysis, we excluded 2620 participants with cognitive impairment to avoid the occurrence of oversized recall bias when answering the question. In the third sensitivity analysis, 339 participants who lived in their current location for less than or equal to one year were excluded from the analysis.

The statistical significance was determined by a two-tailed *P*-value < 0.05. All analyses were performed with STATA version 17.0 (Stata Corp, College Station, TX, USA), R version 3.6.3 (R Foundation for Statistical Computing), and Empower (R) software (www.empowerstats.com,X&Y Solutions, Inc., Boston, MA, USA).

## Results

### Characteristics of participants

The characteristics of the participants are shown in Table 1. And participants had a median age of 85.00 years. The proportion of individuals with chronic multimorbidity is 36.89%. Of the participants, 55.37% were women, and 55.47% lived in urban areas. Individuals residing at a greater distance from a major roadway exhibited a higher probability of being older, living in rural, having no spouse, having a lower level of education, having a lower annual household income, having no physical activity, and having no depression and anxiety symptoms.

**Table 1:** Main characteristics of the study population and its subgroups by residential proximity to major roadways

Characteristics	Residential proximity to major roadways (m)						*P*-value
	Total (*N* = 12,214)	< 50 (*N* = 2242)	50–100 (*N* = 1866)	101–200 (*N* = 1262)	201–300 (*N* = 1153)	> 300 (*N* = 5691)	
Age (years), median (IQR)	85.00 (76.00, 95.00)	84.00 (75.00, 94.00)	84.00 (75.00, 94.00)	85.00 (76.00, 95.00)	85.00 (76.00, 96.00)	85.00 (76.00, 95.00)	0.006
Self-rated health status, median (IQR)	3.00 (3.00, 4.00)	3.00 (3.00, 4.00)	3.00 (3.00, 4.00)	3.00 (3.00, 4.00)	3.00 (3.00, 4.00)	3.00 (3.00, 4.00)	0.036
Chronic multimorbidity, n (%)							< 0.001
Yes	4506 (36.89)	857 (38.22)	769 (41.21)	546 (43.26)	451 (39.12)	1883 (33.09)	
No	7708 (63.11)	1385 (61.78)	1097 (58.79)	716 (56.74)	702 (60.88)	3808 (66.91)	
Gender, n (%)							0.649
Male	5451 (44.63)	990 (44.16)	851 (45.61)	571 (45.25)	495 (42.93)	2544 (44.70)	
Female	6763 (55.37)	1252 (55.84)	1015 (54.39)	691 (54.75)	658 (57.07)	3147 (55.30)	
Area of residence, n (%)							< 0.001
Rural	5439 (44.53)	896 (39.96)	585 (31.35)	423 (33.52)	437 (37.90)	3098 (54.44)	
Urban	6775 (55.47)	1346 (60.04)	1281 (68.65)	839 (66.48)	716 (62.10)	2593 (45.56)	
Ethnicity, n (%)							< 0.001
Han	9846 (93.50)	1836 (92.26)	1625 (94.81)	1096 (95.80)	947 (92.48)	4342 (93.22)	
Other	684 (6.50)	154 (7.74)	89 (5.19)	48 (4.20)	77 (7.52)	316 (6.78)	
Marital status, n (%)							0.422
Have a spouse	5068 (41.91)	958 (43.08)	786 (42.49)	528 (42.11)	453 (39.74)	2343 (41.65)	
Have no spouse	7026 (58.09)	1266 (56.92)	1064 (57.51)	726 (57.89)	687 (60.26)	3283 (58.35)	
Education level, n (%)							< 0.001
0 year	5076 (48.69)	979 (49.75)	743 (43.96)	504 (43.98)	480 (47.52)	2370 (51.39)	
1–6 years	3331 (31.95)	629 (31.96)	484 (28.64)	352 (30.72)	290 (28.71)	1576 (34.17)	
> 6 years	2019 (19.37)	360 (18.29)	463 (27.40)	290 (25.31)	240 (23.76)	666 (14.44)	
Annual household income (yuan), n (%)							< 0.001
< 30,000	7337 (65.39)	1353 (65.39)	1079 (62.59)	734 (64.06)	648 (62.19)	3532 (67.23)	
30,000–50,000	2384 (21.25)	458 (22.14)	352 (20.42)	227 (19.81)	224 (21.50)	1123 (21.43)	
> 50,000	1500 (13.37)	258 (12.47)	293 (17.00)	185 (16.14)	170 (16.31)	594 (11.34)	
Smoking status, n (%)							0.058
Yes	1814 (15.00)	342 (15.46)	271 (14.65)	167 (13.33)	148 (12.99)	886 (15.71)	
No	10,280 (85.00)	1870 (84.54)	1579 (85.35)	1086 (86.67)	991 (87.01)	4754 (84.29)	
Alcohol consumption, n (%)							0.082
Yes	1753 (14.57)	303 (13.78)	253 (13.76)	160 (12.91)	170 (14.99)	867 (15.42)	
No	10,279 (85.43)	1896 (86.22)	1586 (86.24)	1079 (87.09)	964 (85.01)	4754 (84.58)	
Physical activity, n (%)							< 0.001
Yes	3725 (30.95)	785 (35.52)	624 (33.84)	407 (32.80)	379 (33.42)	1530 (27.28)	
No	8312 (69.05)	1425 (64.48)	1220 (66.16)	834 (67.20)	755 (66.58)	4078 (72.72)	
Depression symptoms, n (%)							0.647
Absence	8132 (74.67)	1521 (75.48)	1236 (74.01)	835 (73.31)	784 (75.38)	3756 (74.72)	
Presence	2759 (25.33)	494 (24.52)	434 (25.99)	304 (26.69)	256 (24.62)	1271 (25.28)	
Anxiety symptoms, n (%)							0.081
Absence	9999 (88.71)	1820 (87.29)	1551 (89.55)	1036 (87.72)	948 (88.43)	4644 (89.27)	
Presence	1273 (11.29)	265 (12.71)	181 (10.45)	145 (12.28)	124 (11.57)	558 (10.73)	
Cognitive impairment, n (%)							0.042
Without	9594 (78.55)	1795 (80.06)	1475 (79.05)	1015 (80.43)	891 (77.28)	4418 (77.63)	
With	2620 (21.45)	447 (19.94)	391 (20.95)	247 (19.57)	262 (22.72)	1273 (22.37)	

Abbreviation: IQR: interquartile range

Note: Total percentages within categories may not equal 100% due to rounding

### Association between residential proximity to major roadways and chronic multimorbidity

Residential proximity to major roadways was found to be significantly associated with chronic multimorbidity in the unadjusted logistic regression analysis of Model 1 (Table 2). After adjusting for age, gender, area of residence, ethnicity, and marital status in Model 2, compared with those living over 300 m away, the OR (95% CI) for individuals living at distances of 201-300 m, 101-200 m, 50-100 m, and less than 50 m from major roadways were 1.21 (1.05, 1.39), 1.35 (1.18, 1.54), 1.22 (1.08, 1.37), 1.14 (1.02, 1.27) respectively. After adjusting for all variables in Model 3, a steady pattern resembling that of Model 1 and Model 2 was noted in the association between residential proximity to major roadways and chronic multimorbidity. Compared with those living over 300 m away, the OR (95% CI) for individuals living at distances of 201-300 m, 101-200 m, 50-100 m, and less than 50 m from major roadways were 1.23 (1.04, 1.46), 1.24 (1.06, 1.46), 1.18 (1.03, 1.36), and 1.16 (1.01, 1.32).

**Table 2:** The association between residential proximity to major roadways (m) and chronic multimorbidity

Model	< 50		50–100		101–200		201–300		> 300
	OR (95%CI)	*P*-value	OR (95%CI)	*P*-value	OR (95%CI)	*P*-value	OR (95%CI)	*P*-value	OR (95%CI)
Model 1	1.25 (1.13, 1.39)	< 0.001	1.42 (1.27, 1.58)	< 0.001	1.54 (1.36, 1.75)	< 0.001	1.30 (1.14, 1.48)	< 0.001	Ref.
Model 2	1.14 (1.02, 1.27)	0.025	1.22 (1.08, 1.37)	0.001	1.35 (1.18, 1.54)	< 0.001	1.21 (1.05, 1.39)	0.009	Ref.
Model 3	1.16 (1.01, 1.32)	0.031	1.18 (1.03, 1.36)	0.018	1.24 (1.06, 1.46)	0.008	1.23 (1.04, 1.46)	0.014	Ref.

Abbreviation: OR: Odds ratios, CI: Confidence intervals

Model 1 was unadjusted

Model 2 was adjusted for age, gender, area of residence, ethnicity, and marital status

Model 3 was adjusted for age, gender, area of residence, ethnicity, marital status, education level, annual household income, smoking status, alcohol consumption, physical activity, depression symptoms, anxiety symptoms, cognitive impairment, and self-rated health status

### Stratified and interaction analyses

**Fig. 2:**
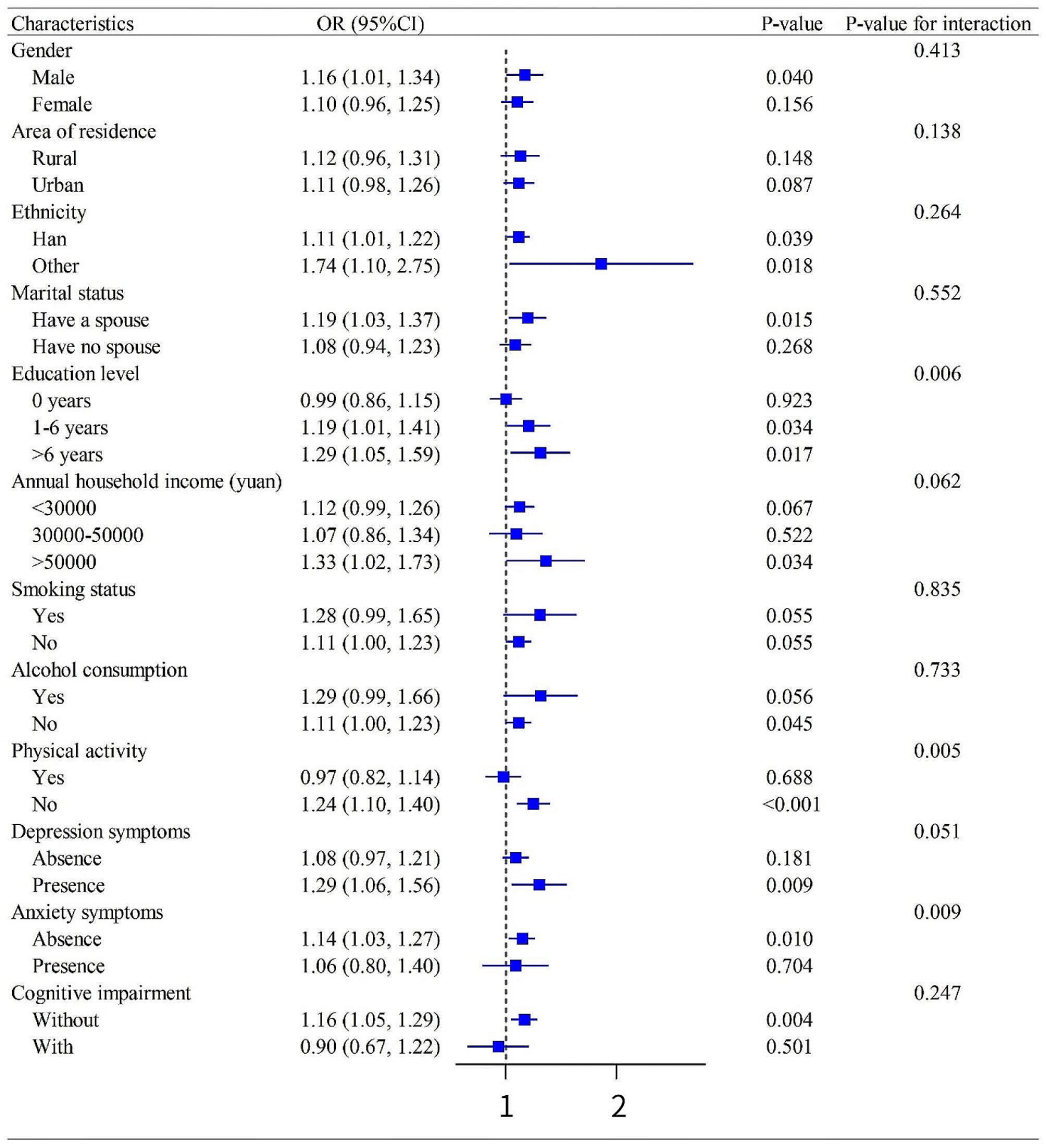
Association of residential proximity to major roadways (≤ 200 m vs. >200 m) with chronic multimorbidity among subgroups. Abbreviation: OR: Odds ratios, CI: Confidence intervals. Adjustments: age, gender, area of residence, ethnicity, marital status, education level, annual household income, smoking status, alcohol consumption, physical activity, depression symptoms, anxiety symptoms, cognitive impairment, and self-rated health status, except for the stratified variables in each subgroup

We conducted subgroup analyses for 12 categorical variables (Fig. 2). The association between residential proximity to major roadways and chronic multimorbidity was persistent and consistent across these subgroups. There was an interaction between dichotomous residential proximity to major roadways and education level (P for interaction = 0.006), physical activity (P for interaction = 0.005), and anxiety symptoms (P for interaction = 0.009). The associations were stronger in those with education levels > 6 years (1.29 [1.05, 1.59], *P* = 0.017) than their counterparts exhibited. Meanwhile, the association of residential proximity to major roadways and chronic multimorbidity was more significant in the group having no physical activity (1.24 [1.10, 1.40], *P* < 0.001) and no anxiety symptoms (1.14 [1.03, 1.27], *P* = 0.010). In contrast, a weaker association was detected in the population that had physical activity and anxiety symptoms.

### Sensitivity analyses

In the first sensitivity analysis, all analyses were repeated after the multiple imputation for the missing values. The outcomes of this analysis stay stable (all *P* < 0.05) (Supplementary Table 1). In the second sensitivity analysis, we assessed the possible effects on outcomes after the exclusion of individuals with cognitive impairment. Notably, the removal of these participants did not change the statistical significance of the associations observed in all analyses (all *P* < 0.05) (Supplementary Table 2). In the third sensitivity analysis, after excluding participants living in their current location for less than or equal to one year, the results stay stable and interpretation remains unchanged (all *P* < 0.05) (Supplementary Table 3).

## Discussion

To our knowledge, this is the inaugural exploration of the association between residential proximity to major roadways and chronic multimorbidity. The robust associations consistently existed even after adjusting for numerous confounding variables. In addition, the association is particularly significant among individuals with a high level of education.

This study identified an association between residential proximity to major roadways and chronic multimorbidity, broadening the understanding of the association between residential proximity to major roadways and older adults’ health status. Previous research has found residential proximity to major roadways was associated with some single chronic diseases, such as hypertension among older adults [13], stroke among adults aged older than 40 years [14], and dementia that occurs from 55 to 85 years [15]. One potential reason for the association of residential proximity to major roadways and chronic multimorbidity is being exposed to air pollution [43]. The characteristics of major roadways are high traffic volume and vehicular emissions, resulting in increased levels of harmful pollutants in the surrounding it, such as particulate matter and nitrogen dioxide. Prolonged exposure to such pollutants is extensively associated with adverse health outcomes, such as respiratory diseases [44], chronic kidney disease [45] and Parkinson’s disease [46]. On the other hand, the closer residential proximity to major roadways, the higher the exposure to traffic noise. Research has demonstrated traffic noise has established a clear link to chronic diseases, such as cardiovascular diseases [47], respiratory diseases [48], and type 2 diabetes [49]. Meanwhile, traffic noise during the night can reduce sleep duration [50], and inadequate sleep duration has been shown to be associated with multimorbidity [51]. As well, studies have indicated that an excessive amount of noise at work may cause employees to produce more catecholamine production, an indicator of elevated stress [52]. Stress is associated with the dysregulation of various physiological systems, including the immune, endocrine, and cardiovascular systems [53]. Such dysregulation can lead to the occurrence and deterioration of chronic diseases, ultimately increasing the incidence of chronic multimorbidity among older adults [54].

The significance of residing far from the main road is highlighted in our research because the selection of house location represents a relatively changeable factor for not only individuals but also the Bureau of Municipal and Rural Construction. Additionally, this highlights the imperative of implementing interventions focusing on public health in regions residential proximity to major roadways, such as the setup of green buffer zones [55] and the installation of sound barriers [56]. Hence, if subsequent research carried out in comparable developing nations were to validate these results, it could greatly impact public health efforts to tackle the growing occurrence of chronic multimorbidity among older adults amidst the backdrop of rapid population aging.

Several biological mechanisms have been enumerated to interpret the reasons and pathways that residential proximity to major roadways may be associated with chronic multimorbidity. Residential proximity to major roadways is a multifaceted exposure. First, traffic-related air pollutants, such as particulate matter and nitrogen dioxide, induce oxidative stress on the respiratory epithelium and cell membranes, producing reactive oxygen species and initiating gene activation [43, 57], thereby triggering an inflammatory reaction, resulting in several chronic diseases, such as cancer [58], asthma [59], and cardiovascular diseases [60]. Simultaneously, traffic-related air pollution can affect the development or worsening of cognitive function [32]. Neuroimaging studies suggest that higher ventricle volumes in those exposed to higher levels of particulate matter coarse indicated increased overall brain atrophy, which could impact cognitive function and lead to dementia [61]. Moreover, air pollution causes the innate immunity in the lung to become activated, and this immune activation then spreads to other organs and tissues and manifests as persistently elevated blood levels of pro-inflammatory biomarkers, which impair insulin sensitivity and beta-cell function and ultimately lead to metabolic diseases, such as diabetes [62]. Second, proximity to major roadways usually causes more serious traffic noise. Exposure to traffic noise can raise stress hormone levels, to increase levels of cortisol, noradrenaline, dopamine, angiotensin II, and endothelin-1 [63]. Additionally, noise exposure induces autonomic imbalance through elevated blood pressure, heart rate, sympathetic activation or parasympathetic withdrawal, and stiffer arteries [64, 65]. Both of which can increase the possibility of cardiometabolic diseases [49, 66–68]. Finally, traffic noise can induce stress [69]. Prolonged exposure to acute and chronic stress can disturb the ideal state of neuroendocrine reactivity, increasing the organism’s susceptibility to stressors, and facilitating a well-established, frequently observed increased likelihood of mental and physical (co-) morbidity [54]. It is imperative to acknowledge that these biological mechanisms mentioned above are not mutually exclusive and may have interdependent effects on the emergence and progression of chronic multimorbidity in residential proximity to major roadways. Four pathways have been proved to better explain the adverse impact of simultaneous air pollution and noise exposure: a dysregulated autonomic nervous system and/or an activated sympathetic system; the production of inflammatory mediators; modifications to lipids or phospholipids; recruitment of various immune cell populations; oxidative impairment of endothelial dysfunction; and increased activity of prothrombotic signaling pathways [70–72]. It can be seen that the mechanisms of the association of residential proximity to major roadways and chronic multimorbidity are complicated.

There was a stronger association between residential proximity to major roadways and chronic multimorbidity among individuals with higher level of education. This result is consistent with a previous study, which found that the association between residential proximity to major roadways and the prevalent hypertension was more pronounced among those with, at minimum, a college degree [73]. However, another study conducted in the Netherlands demonstrated a more significant association between being exposed to air pollution from traffic and the death rate among individuals with lower level of education [74]. This seemingly paradoxical outcome can be attributed to differences in sociocultural contexts. In European countries, individuals with a higher level of education may tend to understand and follow health-promoting methods, thereby attenuating the impact of environmental hazards [75]. In contrast, in other countries, particularly in Asia, although individuals with higher education might understand the hazards mentioned above, their ability to adopt healthy behaviors still may be limited by lifestyle [76] or occupational stress [77]. It has been verified that in China, individuals with a higher level of education are more inclined to prefer residential proximity to major roadways [32], which gives them access to richer social resources and shorter commute times. This result underscores the need to integrate people’s health considerations into traffic control and policy development, especially for individuals with higher education facing heightened risks.

It is important to recognize several limitations. First, as a cross-sectional study, we cannot deduce a causal link between residential proximity to major roadways and chronic multimorbidity among the study population. Second, we cannot exclude the possibility that participants with chronic multimorbidity may choose residential proximity to major roadways for the convenience of seeking medical treatment and shopping. Third, it should be acknowledged that unmeasured confounding variables might have an influence on the observed outcomes. For example, although this study includes geographic variables like residential areas, it lacks comprehensive data on additional environmental characteristics, such as natural elements (proximity to rivers and green spaces), and anthropogenic factors (e.g. polluting companies). Finally, the current residential proximity to the nearest major roadways is derived from self-reported data and is not field measurements, which might increase the recall bias and data inaccuracy.

## Conclusion

This study reveals a robust association between residential proximity to major roadways and chronic multimorbidity. Additionally, a higher level of education will enhance this association. These findings provide support for subsequent research concerning the adverse effects of residential proximity to major roadways on individuals and offer important reference value to the reform of residential area siting, transportation policies, and environmental regulations. For instance, these findings can provide a reference for the selection of older adults’ retirement living locations, the setup of green buffer zones, and the installation of sound barriers to decrease the risk of chronic multimorbidity resulting from traffic-related exposure.

### Electronic supplementary material

Below is the link to the electronic supplementary material.

Supplementary Material 1

## Data Availability

The datasets utilized for this study are available on the Peking University Open Research Data website: https://opendata.pku.edu.cn/dataset.xhtml?persistentId=doi:10.18170/DVN/WBO7LK.
