# Supplementary material for: Association between residential proximity to major roadways and chronic multimorbidity among Chinese older adults: a nationwide cross-sectional study
Source: BMC Geriatr. 2024 Jan 29;24:111. doi: 10.1186/s12877-024-04712-z (PMC10826232; doi:10.1186/s12877-024-04712-z)
Supplement: Supplementary file 1 — Supplementary Material 1 [file 12877_2024_4712_MOESM1_ESM.doc]

**Supplementary Table 1.** Association of residential proximity to major roadways (m) with chronic multimorbidity after multiple imputations.

| Model | <50 | | 50-100 | | 101-200 | | 201-300 | | >300 |
| --- | --- | --- | --- | --- | --- | --- | --- | --- | --- |
|  | OR (95%CI) | *P*-value | OR (95%CI) | *P*-value | OR (95%CI) | *P*-value | OR (95%CI) | *P*-value | OR (95%CI) |
| Model 1 | 1.25 (1.13, 1.39) | <0.001 | 1.42 (1.27, 1.58) | <0.001 | 1.54 (1.36, 1.75) | <0.001 | 1.30 (1.14, 1.48) | <0.001 | Ref. |
| Model 2 | 1.16 (1.04, 1.28) | 0.006 | 1.25 (1.12, 1.40) | <0.001 | 1.39 (1.22, 1.57) | <0.001 | 1.21 (1.06, 1.38) | 0.005 | Ref. |
| Model 3 | 1.19 (1.06, 1.33) | 0.003 | 1.24 (1.10, 1.40) | <0.001 | 1.33 (1.16, 1.52) | <0.001 | 1.23 (1.07, 1.42) | 0.005 | Ref. |

Abbreviation: OR: Odds ratios, CI: Confidence intervals.

Model 1 was unadjusted.

Model 2 was adjusted for age, gender, area of residence, ethnicity, and marital status.

Model 3 was adjusted for age, gender, area of residence, ethnicity, marital status, education level, annual household income, smoking status, alcohol consumption, physical activity, depression symptoms, anxiety symptoms, cognitive impairment, and self-rated health status.

**Supplementary Table 2.** Association of residential proximity to major roadways (m) with chronic multimorbidity excluding participants who suffered from cognitive impairment.

| Model | <50 | | 50-100 | | 101-200 | | 201-300 | | >300 |
| --- | --- | --- | --- | --- | --- | --- | --- | --- | --- |
|  | OR (95%CI) | *P*-value | OR (95%CI) | *P*-value | OR (95%CI) | *P*-value | OR (95%CI) | *P*-value | OR (95%CI) |
| Model 1 | 1.25 (1.12, 1.40) | <0.001 | 1.48 (1.31, 1.67) | <0.001 | 1.60 (1.39, 1.84) | <0.001 | 1.37 (1.19, 1.59) | <0.001 | Ref. |
| Model 2 | 1.15 (1.02, 1.30) | 0.027 | 1.27 (1.11, 1.45) | <0.001 | 1.40 (1.21, 1.63) | <0.001 | 1.26 (1.08, 1.48) | 0.004 | Ref. |
| Model 3 | 1.17 (1.02, 1.35) | 0.022 | 1.23 (1.06, 1.43) | 0.005 | 1.29 (1.09, 1.53) | 0.003 | 1.25 (1.05, 1.50) | 0.013 | Ref. |

Abbreviation: OR: Odds ratios, CI: Confidence intervals.

Model 1 was unadjusted.

Model 2 was adjusted for age, gender, area of residence, ethnicity, and marital status.

Model 3 was adjusted for age, gender, area of residence, ethnicity, marital status, education level, annual household income, smoking status, alcohol consumption, physical activity, depression symptoms, anxiety symptoms, and self-rated health status.

Supplementary Table 3. Association of residential proximity to major roadways (m) with chronic multimorbidity excluding participants living in their current location for less than or equal to one year.

| Model | <50 | | 50-100 | | 101-200 | | 201-300 | | >300 |
| --- | --- | --- | --- | --- | --- | --- | --- | --- | --- |
|  | OR (95%CI) | *P*-value | OR (95%CI) | *P*-value | OR (95%CI) | *P*-value | OR (95%CI) | *P*-value | OR (95%CI) |
| Model 1 | 1.25 (1.13, 1.38) | <0.001 | 1.42 (1.27, 1.58) | <0.001 | 1.57 (1.38, 1.78) | <0.001 | 1.31 (1.15, 1.50) | <0.001 | Ref. |
| Model 2 | 1.13 (1.01, 1.27) | 0.030 | 1.23 (1.09, 1.38) | 0.001 | 1.38 (1.20, 1.58) | <0.001 | 1.23 (1.06, 1.42) | 0.005 | Ref. |
| Model 3 | 1.15 (1.01, 1.32) | 0.034 | 1.21 (1.05, 1.39) | 0.009 | 1.28 (1.09, 1.50) | 0.003 | 1.24 (1.05, 1.47) | 0.012 | Ref. |

Abbreviation: OR: Odds ratios, CI: Confidence intervals.

Model 1 was unadjusted.

Model 2 was adjusted for age, gender, area of residence, ethnicity, and marital status.

Model 3 was adjusted for age, gender, area of residence, ethnicity, marital status, education level, annual household income, smoking status, alcohol consumption, physical activity, depression symptoms, anxiety symptoms, cognitive impairment, and self-rated health status.
